# Machine Learning-based Classification of Diffuse Large B-cell Lymphoma Patients by Their Protein Expression Profiles[Fn FN1]

**DOI:** 10.1074/mcp.M115.050245

**Published:** 2015-08-26

**Authors:** Sally J. Deeb, Stefka Tyanova, Michael Hummel, Marc Schmidt-Supprian, Juergen Cox, Matthias Mann

**Affiliations:** From the ‡Proteomics and Signal Transduction Group and; §Computational Systems Biochemistry, Max Planck Institute of Biochemistry, D-82152 Martinsried, Germany,; ¶Institute of Pathology, Campus Benjamin Franklin, Molecular Diagnostics, Charité-Universitätsmedizin Berlin, 12200 Berlin, Germany, and; ‖Institute of Oncology and Hematology, III. Medizinische Klinik, Technische Universität München, 81675 Munich, Germany

## Abstract

Characterization of tumors at the molecular level has improved our knowledge of cancer causation and progression. Proteomic analysis of their signaling pathways promises to enhance our understanding of cancer aberrations at the functional level, but this requires accurate and robust tools. Here, we develop a state of the art quantitative mass spectrometric pipeline to characterize formalin-fixed paraffin-embedded tissues of patients with closely related subtypes of diffuse large B-cell lymphoma. We combined a super-SILAC approach with label-free quantification (hybrid LFQ) to address situations where the protein is absent in the super-SILAC standard but present in the patient samples. Shotgun proteomic analysis on a quadrupole Orbitrap quantified almost 9,000 tumor proteins in 20 patients. The quantitative accuracy of our approach allowed the segregation of diffuse large B-cell lymphoma patients according to their cell of origin using both their global protein expression patterns and the 55-protein signature obtained previously from patient-derived cell lines (Deeb, S. J., D'Souza, R. C., Cox, J., Schmidt-Supprian, M., and Mann, M. (2012) *Mol. Cell. Proteomics* 11, 77–89). Expression levels of individual segregation-driving proteins as well as categories such as extracellular matrix proteins behaved consistently with known trends between the subtypes. We used machine learning (support vector machines) to extract candidate proteins with the highest segregating power. A panel of four proteins (PALD1, MME, TNFAIP8, and TBC1D4) is predicted to classify patients with low error rates. Highly ranked proteins from the support vector analysis revealed differential expression of core signaling molecules between the subtypes, elucidating aspects of their pathobiology.

Clinical differences between human cancer subtypes have long been recognized by oncologists. However, comprehensive analyses of the underlying molecular differences have only become possible with the recent advent of powerful oligonucleotide-based technologies that allow global profiling of individual tumors (1). The potential benefits of improved molecular characterization are enormous (2). In fact, the molecular understanding of tumorigenesis and cancer progression is promising to enable a shift from nonspecific cytotoxic drugs to drugs that are much more targeted toward cancer cells. An important step to achieve targeted therapies is to reliably identify the group of patients that are likely to benefit from a specific drug or treatment strategy. This ability to group cancer patients into clinically meaningful subtypes is a challenging task that requires well designed and robust approaches.

More than a decade ago, gene expression profiling discovered two subtypes of diffuse large B-cell lymphoma (DLBCL)^1^ that are morphologically indistinguishable (3). The subtyping was based on gene expression signatures that correspond to stages of B-cell development from which the tumor is derived. The germinal center B-cell-like DLBCL (GCB-DLBCL) transcriptome was dominated by genes characteristic of germinal center B-cells, whereas the transcriptome of activated B-cell-like DLBCL (ABC-DLBCL) more closely resembled activated B-cells *in vitro* (3). Importantly, the discovered subtypes defined prognostic categories (3, 4), opening up the possibility of differential treatment (5). Nonetheless, this cell-of-origin (COO) classification did not fully reflect the differences in overall survival after chemotherapy among patients. Follow-up studies (also using gene expression profiling) showed that a multivariate model constructed from three gene expression signatures (germinal center B-cell, stromal-1, and stromal-2) was a better predictor of survival (6). Stromal-1 reflected extracellular matrix deposition, and stromal-2, which had an unfavorable prognosis, reflected tumor blood vessel density.

In addition to DLBCLs, gene expression profiling also successfully subclassified several other cancer types such as breast cancer (7). However, in colorectal adenocarcinoma, there was no correlation between the subtypes derived from gene expression profiling and clinical phenotypes like patient survival and response to treatment (8). As RNA is a fragile molecule, one of the challenges of mRNA-based global expression studies is the required quality of the RNA sample (9). The problem is exacerbated when working with formalin-fixed paraffin-embedded (FFPE) tissues, which are frequently the only biopsy material available. The extraction of RNA from FFPE tissues is still a difficult task, and snap frozen tissues are preferred for microarray-based genome-wide gene expression profiling (10). For that reason and because proteins are established markers in immunohistopathology, in the last decade, many approaches were developed to classify DLBCL patients on the basis of immunohistochemistry of FFPE tissues. They attempted to simulate gene expression profiling in predicting the COO of tumors. However, gene expression profiling rather than immunohistochemistry-based algorithms still best predicted prognosis in DLBCL patients treated with immunochemotherapy (11). Most recently, a targeted RNA (NanoString)-based test of 20 genes accurately assigned COO subtypes to DLBCL patients using FFPE tissue (12) and has now been adopted as a diagnostic tool in a clinical trial to support the development of lenalidomide (Revlimid) as treatment for patients with DLBCL.

Proteins are the molecules that carry out essentially all biological functions in a cell. Thus, proteomics has the potential to directly assess deregulated cellular processes and signaling pathways. In the last decade, MS-based proteomics has developed tremendously in terms of sample preparation techniques, mass spectrometric instrumentation, and data analysis. Enhanced sensitivity, accuracy, and peptide sequencing speed of contemporary mass spectrometers allow the identification of thousands of proteins in a single experiment. This has already resulted in almost the complete coverage of complex biological samples such as human cancer cells (13, 14). We have shown that very large depth of complex proteomes can even be attained without prefractionation (single shot measurements) (15, 16). In addition, proteins and their post-translational modifications can be efficiently extracted from FFPE tissues (17). There have been complementary, enormous advances in data analysis and data management tools, facilitating the wide adoption of MS-based proteomics. In particular, these developments mean that characterizing small cohorts of human cancer patients in a reasonable amount of time is finally becoming feasible.

Previously, we have successfully subtyped DLBCL cell lines on the basis of their total protein expression patterns (18) and on their *N*-glycosylated peptide patterns (19). In this study, we decided to explore the applicability of our high resolution MS-based platform to the problem of cancer subtyping from macrodissected slices of FFPE tissue from patient samples. For quantification, we took advantage of the high accuracy of the super-SILAC approach (20) and combined it with label-free quantification of the proteins not present in the spiked-in standard. In addition to segregating cancer subtypes by our previously derived 55-protein signature and by the total protein expression patterns, we derived a novel combination of statistical feature selection and machine learning to define a small signature of differentiating proteins with the highest segregating power. This analysis also allowed us to dissect important molecular differences between the subtypes.

## EXPERIMENTAL PROCEDURES

### 

#### 

##### Generation of the Lymphoma super-SILAC Mix

The super-SILAC mix was generated by combining equal amounts of heavy lysates from six lymphoma cell lines (Ramos, Mutu, BL-41, U2932, L428, and DB) as described (18). Stocks of this mix were prepared and used as standards that were spiked in each of the cell lines we previously studied and the 20 patient samples we analyzed in this study.

##### FFPE Human Tissues

FFPE samples of DLBCL were obtained from the Institute of Pathology, Charité-Universitätsmedizin Berlin. Analysis of the samples was approved by the local ethics committee (registration number EA4/085/07).

##### Protein Extraction from FFPE DLBCL Tissues

For each patient sample, two FFPE slices of macrodissected tissue were collected (10-μm thickness). They were processed for mass spectrometry-based proteome analysis by extraction and digestion according to the filter-aided sample preparation (FASP) protocol (FFPE-FASP) (17, 21). In short, FFPE tissue slices were incubated in 1 ml of xylene (two times) with gentle agitation for 5 min at room temperature. After removing the paraffin, the samples were dried by incubating them in 1 ml of absolute ethanol (two times). The dried samples were then lysed in a buffer consisting of 0.1 m Tris-HCl (pH 8.0), 0.1 m DTT, and 4% sodium dodecyl sulfate (SDS). After sonification, the samples were boiled at 99 °C using a heating block with agitation (600 rpm) for 60 min. The samples were then cleared by centrifugation.

##### Protein Digestion and Peptide Fractionation

On a 30-kDa filter (Millipore, Billerica, MA), 100 μg of each of the patient samples and the super-SILAC mix were mixed. The samples were further processed by the FASP method in which the SDS buffer is exchanged with a urea buffer (21). This was followed by alkylation with iodoacetamide and overnight digestion by trypsin at 37 °C in 50 mm ammonium bicarbonate. The tryptic peptides were collected by centrifugation and elution with water (two times).

Strong anion exchange chromatography was used to fractionate 40 μg of peptides from each patient sample (22). It was performed in tip-based columns from 200-μl micropipette tips stacked with six layers of a 3M Empore anion exchange disk (1214-5012; Varian, Palo Alto, CA). For the fractionation, a Britton and Robinson universal buffer (20 mm acetic acid, 20 mm phosphoric acid, and 20 mm boric acid) was used and titrated using NaOH to six buffers with the desired pH values (pH 11, 8, 6, 5, 4, and 3). Subsequently, six fractions from each sample were collected followed by desalting the eluted fractions on reversed phase C_18_ Empore disc StageTips (23). The peptides were eluted from the StageTips using 20 μl of buffer B composed of 80% ACN in 0.5% acetic acid (two times). A SpeedVac concentrator was used to prepare the samples for MS analysis by removing the organic solvents.

##### LC-MS/MS Analysis

Peptides were separated by nanoflow HPLC (Thermo Fisher Scientific) coupled on line to a quadrupole Orbitrap mass spectrometer (Q Exactive, Thermo Fisher Scientific) with a nanoelectrospray ion source. The peptides were eluted at a flow rate of 200 nl min^−1^ on an in house-made C_18_ reversed phase column that was 50 cm long with a 75-μm inner diameter and packed with ReproSil-Pur C_18_-AQ 1.8-μm resin (Dr. Maisch GmbH, Ammerbuch-Entringen, Germany) in buffer A (0.5% acetic acid). For optimal separation based on average peptide hydrophobicity, four different linear gradients over a period of 205 min were applied. For pH 11 fraction, a gradient of 2–25% buffer B was used; for pH 8 fraction, a gradient of 7–25% buffer B was used; for pH 6 and 5 fractions, a gradient of 7–30% buffer B was used; and for pH 4 and 3 fractions, a gradient of 7–37% buffer B was used. Each gradient was followed by column washing reaching 95% B and then re-equilibration with buffer A.

A data-dependent “top 10” method in which the 10 most abundant precursor ions were selected for fragmentation was used to acquire the data. For survey scans (mass range, 300–1,750 Th), the target value was 3,000,000 with a maximum injection time of 20 ms and a resolution of 70,000 at *m*/*z* 400. An isolation window of 1.6 Th was used for higher energy collisional dissociation with normalized collision energies of 25. For MS/MS scans, the target ion value was set to 1,000,000 with a maximum injection time of 60 ms, a resolution of 17,500 at *m*/*z* 400, and dynamic exclusion of 25 s. This led to a constant injection time of 60 ms, which is fully in parallel with transient acquisition of the previous scan, ensuring fast cycle times.

The patient samples were received in two batches of 10 each that were acquired with the same MS methods. For MS/MS in the second batch, a data-dependent “top 5” method was used where the five most intense ions from the survey scan were selected with an isolation window of 2.2 Th and dynamic exclusion of 45 s. The target ion value was set to 100,000 with a maximum injection time of 120 ms and a resolution of 17,500 at *m*/*z* 400.

##### Data Analysis

We used the MaxQuant software environment (version 1.4.3.9) to analyze MS raw data. The MS/MS spectra were searched against the UniProt database (81,213 entries; release, 2012) using the Andromeda search engine incorporated in the MaxQuant framework (24, 25). Cysteine carbamidomethylation was set as a fixed modification, and N-terminal acetylation and methionine oxidation were set as variable modifications. The maximum false discovery rate for both peptide and protein identifications was set to 0.01. Strict specificity for trypsin cleavage was required allowing N-terminal cleavage to proline. The minimum required peptide length was seven amino acids with a maximum of two miscleavages allowed. The initial precursor mass tolerance was 4.5 ppm, and for the fragment masses, it was up to 20 ppm. The time-dependent recalibration algorithm of MaxQuant was used to improve the mass accuracy of precursor ions. The “match between runs” option was enabled, allowing the matching of identifications across measurements. Relative quantification of the peptides against their SILAC-labeled counterparts was performed with MaxQuant using a minimum ratio count of 1. We combine SILAC with label-free analysis (“hybrid algorithm”) using a minimum count of 1 (see “Results and Discussion”). The bioinformatics analysis was entirely performed using our in house-developed and freely available software Perseus. The data were first filtered to 75% valid values (15 of 20). Missing values were supplied by “data imputation” (width = 0.3, downshift = 1) to simulate signals of low abundance proteins under the assumption that they are biased toward the detection limit of the MS measurement (18). Finally, the data were normalized using width adjustment, which subtracts the median and scales all values in a sample to have equal interquartile range.

##### Principal Component Analysis

Principal component analysis (PCA) was performed on the processed data. In PCA, relying on singular value decomposition, the original feature (protein) space is orthogonally transformed into a set of linearly uncorrelated variables (principal components) that account for different types of variability in the data. In our data set, the source of variability, depicted in components 1 and 4, reflects the molecular difference between the two lymphoma subtypes as measured by the protein profiles.

##### Enrichment Analysis

The enrichment analysis of cancer module categories (26) in the PCA components is based on standard Fisher exact tests (computing the probability of observing exactly this distribution of proteins associated with a particular cancer module (CM) between component 1 and all other components). We apply a multiple hypothesis testing correction using the Benjamini-Hochberg procedure. The significance cutoff was 0.05.

The 2D annotation enrichment procedure is described in detail in Cox and Mann (27). Briefly, a category of proteins (*e.g.* proteins associated with a particular cancer module) is tested for specific expression preferences as compared with the entire distribution of protein expression values. The analysis uses the non-parametric Wilcoxon-Mann-Whitney test that uses rank sums and is further generalized to the analysis of multiple dimensions. An enrichment-specific score is computed that indicates whether the category is enriched for high expression (the score is close to 1) or for low expression (the score is close to −1) values. Comparison of two dimensions simultaneously highlights categories that are similar or different between the lymphoma subtypes.

##### Supervised Learning

In supervised learning, a set of training examples with known labels (in the current data set the samples with known lymphoma subtypes) is used to extract rules from the data based on which two groups can be distinguished. We use support vector machines (SVMs), a technique based on the concept of decision planes that define the boundaries between two groups. The decision planes are determined by the so-called support vectors, which correspond to the samples that are most difficult to distinguish between the subtypes and lie on the margins of the separation plane. In this study, a predictor is trained on the protein expression profiles of patient samples to distinguish between the two lymphoma subtypes. The identification of subtype-characteristic proteins is based on a feature selection technique that requires the proteins to be ranked according to their discriminative power. In particular, we rank the proteins according to the *p* values computed from the modified test statistic (28). The S0 parameter introduces a background correction to improve the signal to noise ratio especially in the case of proteins of low abundance. This approach assigns better ranks to proteins with larger mean-fold changes between the subtypes. To ensure the widest applicability of the results, both the predictor training and the feature selection are done in a cross-validation procedure. This means that the data set is split into training and test subsets multiple times with feature selection and predictor training performed only on the training set. The cross-validation was performed using random sampling with 90% of the data for training and 1,000 repetitions.

## RESULTS AND DISCUSSION

### 

#### 

##### Workflow for Quantitative Proteome Measurements of DLBCL FFPE Patient Samples

One of the most commonly used methods for tissue preservation involves fixing the sample in neutral buffered formalin followed by embedding it in paraffin, termed FFPE tissues. It is routinely used in tissue banks because of its compatibility with immunohistochemistry assays and its long term preservation benefits in an economical format. However, FFPE cohorts have been challenging to use in gene expression studies due to the difficulty to isolate nucleic acids (29). Despite attempts to improve the quality of extracted RNA samples from FFPE tissues and to provide standardized protocols, currently snap frozen tissues are greatly preferred in that workflow (10, 29). In clinical practice, tissue banks of frozen specimens are used for initial discovery studies, but by far the largest sample numbers and almost all tumor specimens are fixed in formalin. Taking advantage of the stability and ease of handling of proteins, we and others have recently shown that protein extraction from FFPE material is possible in a robust manner (17, 30, 31). We did not observe quantitative or qualitative differences between FFPE and frozen tissues at the level of proteins or post-translational modifications (17). Our approach combined boiling in SDS with the FASP method (21). The boiling step presumably reverses the cross-links induced upon fixation, whereas the FASP method allows MS analysis of proteomic samples solubilized in high concentrations of SDS, which is advantageous for FFPE samples (30).

**Fig. 1.**
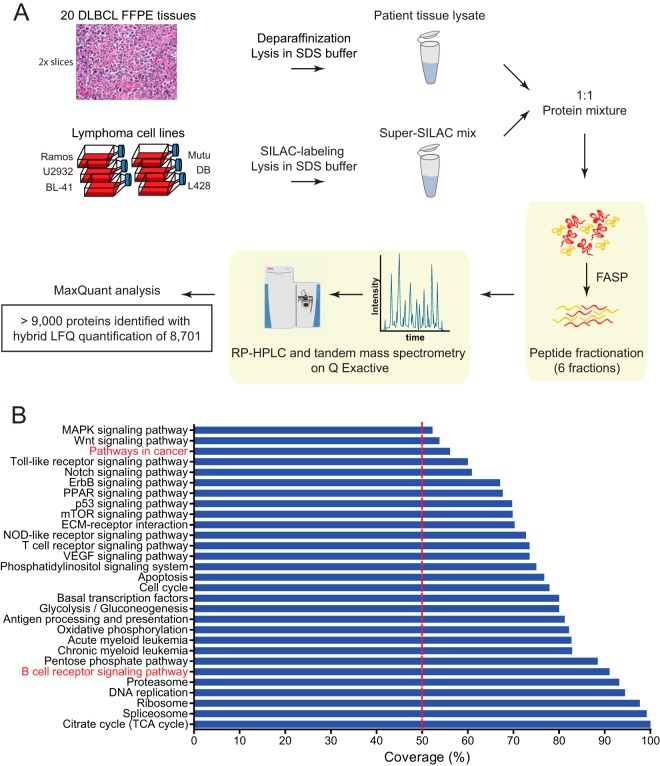
**Proteomic workflow and coverage of 20 FFPE tissue samples from DLBCL patients.** *A*, two slices of macrodissected patient FFPE tissues were processed according to the FASP-FFPE protocol. The super-SILAC approach was used for quantitative measurements using a quadrupole Orbitrap mass spectrometer (Q Exactive). Quantification was based on SILAC ratios combined with label-free quantifications in cases where no SILAC pairs were detected. The data were analyzed using MaxQuant software, resulting in the identification of more than 9,000 proteins. *B*, percent coverage of signaling pathways and cellular processes in the quantified proteomes of DLBCL patients. *RP*, reversed phase; ECM, extracellular matrix; *TCA*, tricarboxylic acid.

Here we macrodissected two slices from each of 20 FFPE tumor samples from DLBCL patients (Fig. 1*A*). Peptides resulting from FASP preparation were subjected to six-step fractionation using a strong anion exchange chromatography protocol followed by LC-MS analysis of each fraction (see “Experimental Procedures”).

Accurate quantification is a requirement for the comparison of the protein expression profiles of the patient samples. For the 20 patient samples, we used our published heavy labeled super-SILAC mix of six lymphoma cell lines optimized to cover a maximal number of “lymphoma-related” proteins (18). Heavy lysates from each of the six cell lines were pooled and spiked in a 1:1 ratio into each of the patient samples. To also quantify SILAC singlets for which the peptide is not found in the reference proteome but is seen in the samples, we introduce a new quantification algorithm in MaxQuant. This so-called hybrid quantification algorithm is a generalization of the MaxLFQ algorithm for the accurate relative quantification of label-free data (32). The essence of the relative quantification step in MaxLFQ is that for each protein and for each sample pair the ratio is calculated for those peptide features that were determined in both samples. In the hybrid quantification algorithm, one distinguishes the case in which a SILAC ratio to the reference is calculated in both samples for a given peptide feature from the case in which one or both ratios cannot be calculated. If both ratios are available, the ratio of ratios is used as input for the MaxLFQ quantification algorithm. In the other case and given that intensities are calculated in both samples for the light SILAC state, the ratio of these light intensities is taken. If one or both light intensities are absent, the peptide feature does not take part in the quantification. All other steps of the MaxLFQ algorithm are applied in exactly the same way in the hybrid LFQ algorithm as well. The result of the hybrid algorithm is an intensity profile for each protein group over all samples, similar to the output of the conventional MaxLFQ algorithm. The whole intensity profile for a protein group can be multiplied with an arbitrary factor because only the relative intensity information is defined by the algorithm.

Combined analysis of the raw MS data by MaxQuant resulted in the identification of 9,012 protein groups across the 20 patient samples (supplemental Table S1). We obtained quantitative results for 8,701 protein groups after using the hybrid LFQ algorithm with an average of 6,278 protein groups in each of the 20 DLBCL patient samples. The average gain from the hybrid LFQ is 353 additional quantifications per sample compared with using SILAC ratios alone (supplemental Fig. S1). This relatively small percentage indicates that the vast majority of proteins were adequately quantifiable against the super-SILAC standard. To investigate the nature of the proteins that we gained from the hybrid LFQ, we performed enrichment analysis based on UniProt keywords on these proteins. Taking sample TRR003 as an example, the two categories with highest significance and an enrichment factor greater than 5 are secreted proteins (FDR = 9.4E−91, enrichment factor = 6.4) and extracellular matrix proteins (FDR = 6.6E−35, enrichment factor = 8.6). Proteins involved in the 3D architecture of tissues in the patient samples and absent in cell lines, readily explain this finding.

##### General Characteristics of the Proteome of 20 DLBCL FFPE Patient Samples

The achieved depth of the proteome resulted in good quantitative coverage of many signaling pathways and cellular processes that play a role in the development and progression of various cancers (Fig. 1*B*). These include processes such as DNA replication (94% coverage of annotated members) and apoptosis (77%). Importantly, there is almost complete coverage (91%) of the B-cell receptor signaling pathway, which can play a major role in lymphomagenesis, and high coverage of other blood cancer-associated proteins such as acute myeloid leukemia (83%) and chronic myeloid leukemia (83%).

**Fig. 2.**
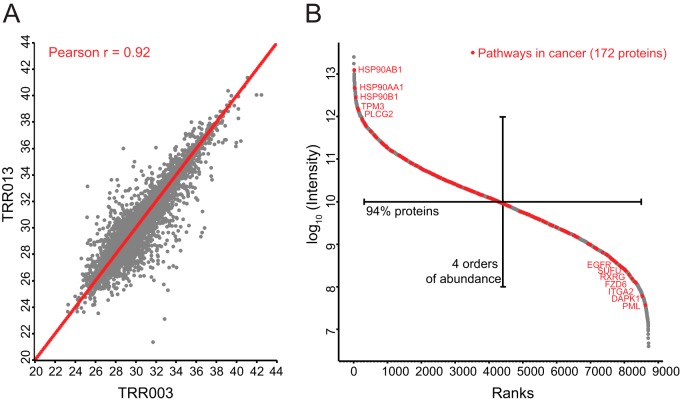
**Quantified proteomes of FFPE tissues from DLBCL patients.** *A*, Pearson's correlation coefficient (*r*) of two representative patient samples (TRR003 and TRR013). *B*, dynamic range of proteomes of DLBCL patients highlighting KEGG annotated proteins involved in *pathways in cancer*.

Pairwise comparisons of all the samples against each other resulted in high Pearson coefficients between the samples (average *r* = 0.92), indicating both high quantitative accuracy between tumor measurements and high similarity in the global proteomes (see Fig. 2*A* for an example). The dynamic range of MS signals for proteins from the patient sample proteomes spanned 7 orders of magnitude with 94% of the proteins concentrated in 4 orders of magnitude (Fig. 2*B*). Overlaying 172 proteins that are annotated in the Kyoto Encyclopedia of Genes and Genomes (KEGG) database as belonging to *pathways in cancer* showed that cancer-related proteins spanned the entire dynamic range. This suggests that both highly and lowly abundant proteins can be important players in DLBCL (Fig. 2*B*).

**Fig. 3.**
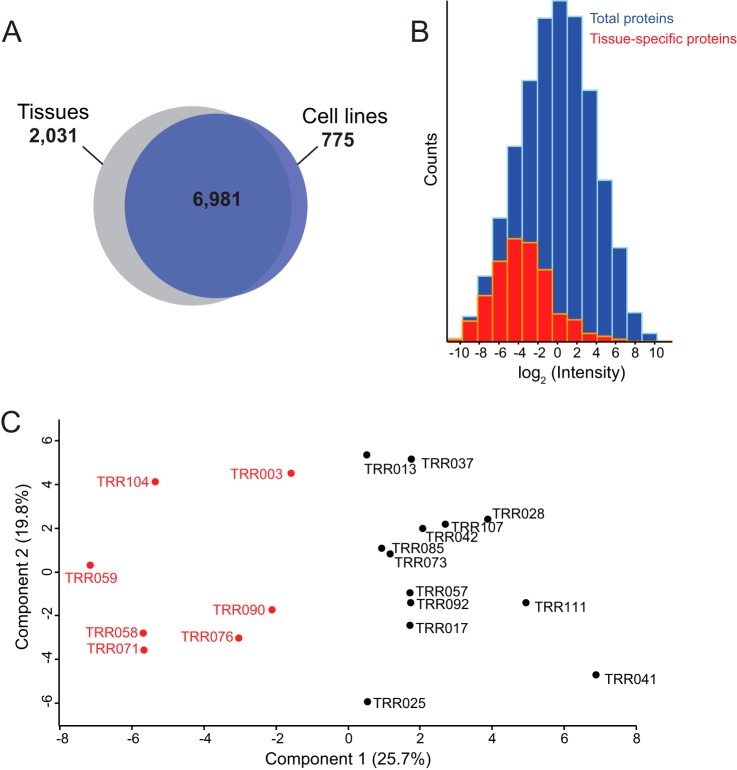
**DLBCL patient tissue proteomes *versus* DLBCL cell line proteomes.** *A*, overlap in protein groups between patient tissue proteomes and cell line proteomes. *B*, the distribution of proteins exclusively quantified in the patient samples (*red*) in comparison with the total distribution (*blue*). *C*, principal component analysis of patient tissue samples using the 55-protein segregating signature derived from cell lines.

Compared with the cell line system we analyzed previously, we detected 2,031 additional protein groups in the present analysis (Fig. 3*A*). We attribute this to technical factors, mainly the very fast and sensitive quadrupole Orbitrap used in this study (33), in combination with the larger complexity of the patient samples. This interpretation is supported by the abundance distribution of the extra 2,031 protein groups, which was at the lower end of the total distribution (Fig. 3*B*). Furthermore, a Fisher exact test for this set of proteins showed the most significant enrichment for proteins located in the *extracellular region part* (FDR = 1.06E−71). This is especially interesting as stromal signatures have already been shown to be important in lymphoma classification (6).

##### The 55-protein Cell Line-derived Signature Correctly Classifies Patients

We previously derived a signature of 55 proteins that robustly segregated ABC-DLBCL and GCB-DLBCL cell lines (18). In addition to proteins that correlated to underlying known biological differences between the subtypes, the cell line signature also included new interesting candidates. To explore the potential of applying this signature to patients, we used the COO subtypes previously established by gene expression profiles on these samples (34). Matching the signature to the patient proteomes after filtering for 75% valid values resulted in quantitative values of 49 proteins in all of the patients. Remarkably, a PCA of these matches clearly segregated the two subtypes (Fig. 3*C*). Thus, our previous proteomic signature can directly be translated to patient samples and classify them correctly although it was derived entirely from a cell line-based system. The loadings of component 1, which accounts for 25.7% of the variability in this small subset of proteins, drive the correct segregation. However, this does not necessarily mean that the cell line signature is optimal to segregate the subtypes with the best possible accuracy. With the increased depth and faithfulness of the patient samples, a signature extracted from the patient proteomes themselves is worth investigating and evaluating as addressed below.

##### Unsupervised Segregation of Patient Samples Based on Their Global Protein Expression Profiles

**Fig. 4.**
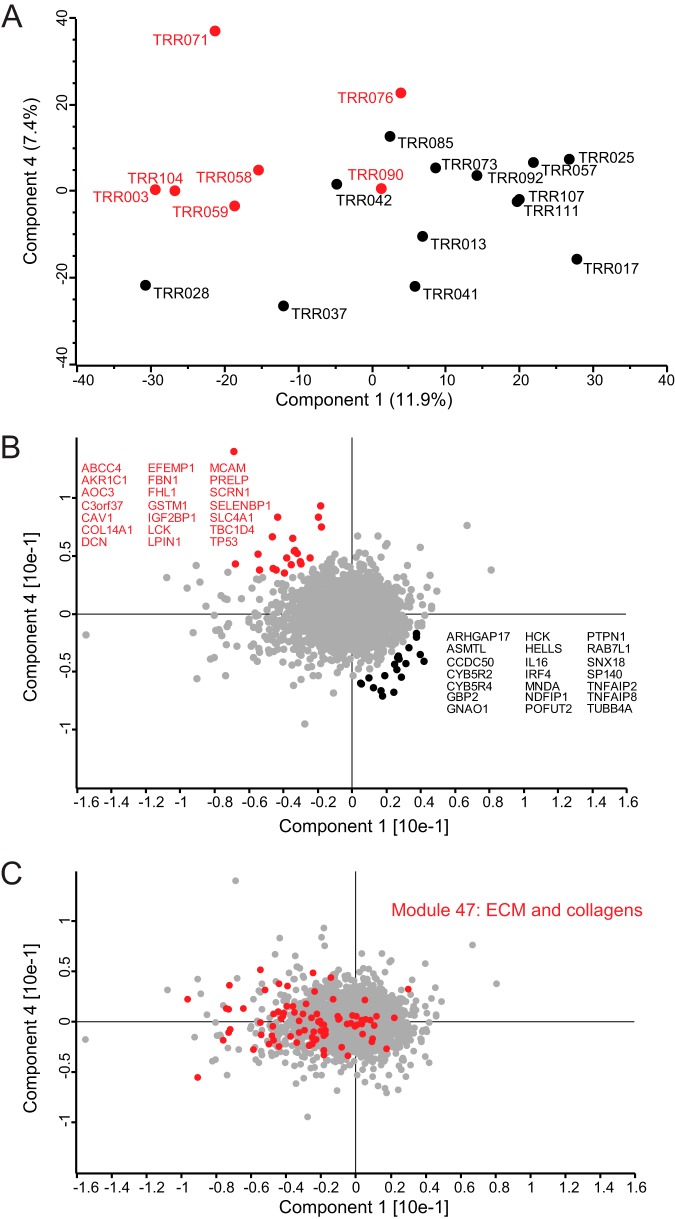
**Principal component analysis of patient samples using their global protein expression profiles.** *A*, the global proteomes of 20 DLBCL patient samples segregated diagonally into ABC-DLBCL (13 samples) and GCB-DLBCL subtypes (seven samples) based on component 1, which accounts for 11.9% of variability, *versus* component 4, which accounts for 7.4% of the variability. *B*, loadings of *A* highlighted in *red* reveal the main proteins driving the COO diagonal segregation. *C*, cancer module 47, which is composed of extracellular proteins and collagens, is highly enriched in component 1.

To explore whether the global protein expression profiles of the patient samples would reveal intrinsic biological differences between the subtypes such as their different COO, we performed a principal component analysis based on the entire protein expression profile of each patient. As performed previously, we filtered for 75% valid values resulting in 5,480 protein groups quantified across the 20 patients. Components 1 *versus* 4 in the PCA provided a diagonal segregation of the patient samples according to their COO classification (Fig. 4*A*). The loadings of such a PCA reveal the drivers causing the segregation (Fig. 4*B*). Among the proteins that are relatively up-regulated in ABC-DLBCL are PTPN1 (PTP1B), IRF4, CCDC50 (Ymer), MNDA, SP140, IL16, RAB7L1, HCK, TNFAIP8, TNFAIP2, and HELLS. Reassuringly, many of these candidates reflect known biological differences between the subtypes. Strong drivers of segregation such as PTPN1, IRF4, and CCDC50 as well as metabolic enzymes such as ARHGAP17 and CYB5R2 were already present in our previously derived cell line signature. This explains the applicability of the cell line-derived signature to segregate patient tissue proteomes and independently confirms the importance of these markers because they were picked up in two independent studies. For instance, IRF4, one of the strong drivers that we previously highlighted, is a transcription factor that drives plasmacytic differentiation, and its expression is directly regulated by NF-κB signaling, a pathogenic hallmark of ABC-DLBCL (35). A new drug (lenalidomide), which inhibits IRF4, selectively kills ABC-DLBCL cells and is currently in clinical trials (36).

The strongest drivers also include some interesting new candidates. One that is up-regulated in ABC-DLBCL is SP140, an interferon-inducible, nuclear lymphocyte-specific protein of unknown function. It is expressed in all human mature B-cells and plasma cell lines as well as in some T-cells (37, 38). It possesses several chromatin-related modules, which suggests a role of SP140 in chromatin-mediated regulation of gene expression (39). A genome-wide association study of single nucleotide polymorphisms for chronic lymphocytic leukemia (CLL) showed that SP140 is a CLL risk locus. Interestingly, that study also identified IRF4 as another risk locus of six loci in total (40). Myeloid cell nuclear differentiation antigen (MNDA) is another strong driver that emerged from the patient data. As the name indicates, MNDA is expressed constitutively in cells of the myeloid lineage, but it can also be expressed by normal and neoplastic B-lymphocytes (41, 42). In a recent study that identified MNDA as a marker for nodal marginal zone lymphoma, the authors also analyzed the expression of MNDA in a cohort of 75 DLBCL cases. Interestingly, of 34 cases in which it was highly expressed, 25 were of the ABC subtype (43). A highly interesting and novel segregator is IL16, a cytokine that is typically characterized as a chemoattractant of CD4^+^ cells to sites of inflammation. Recent studies have suggested an important role of both the promolecule and the secreted form of IL16 in the regulation of lymphocytic cancer cell proliferation (44). In fact, targeting IL16 may be a novel therapeutic approach for cutaneous T-cell lymphoma and multiple myeloma. In multiple myeloma, inhibition of IL16 production by siRNA or IL16 bioactivity by neutralizing antibodies reduces cell proliferation by more than 80% (44).

On the other side of the diagonal segregation are drivers with higher protein levels in the GCB-DLBCL subtype. These include ABCC4, TBC1D4, LCK, CAV1, C3orf37 (HMCES), IGF2BP1, and TP53. TBC1D4 is a Rab GTPase-activating protein that promotes insulin-induced glucose transporter GLUT4 translocation to the plasma membrane, thus increasing glucose uptake (45). TBC1D4 has not yet been associated with lymphoma classification but may be related to increased glucose uptake as observed in many cancer types and may indicate a difference between the cancer types in this respect (46). LCK is a lymphocyte cell-specific protein-tyrosine kinase studied extensively in the context of T-cells where it plays an important role in signal transduction after antigen binding. Dysregulation of LCK expression or LCK kinase activity has been implicated in human and murine T-cell leukemia (47). LCK expression has also been reported in normal B-1 cells and in CLL B-cells (48). It plays an important role in B-cell receptor signaling in CLL, and specific LCK inhibitors have been suggested in the treatment of progressive CLL (49). Reassuringly, LCK has been shown to be present at high levels in normal germinal center cells (50). In addition, it was shown to be expressed in most lymphomas of germinal center origin (*e.g.* follicular lymphoma) and many mantle cell lymphomas, CLL, and most T-cell neoplasms (50).

The diagonal segregation of the subtypes suggested that other biological factors compromised a more clear-cut COO segregation of the patients in the PCA. Enrichment analysis of protein categories showed that *extracellular matrix region part* is one of the strongest gene ontology cellular component (GOCC) categories significantly enriched in component 1 of the PCA (FDR = 1.89E−33). Cancer module (CM) categories correspond to gene sets that are significantly changed in a variety of cancer conditions after mining a large compendium of cancer-related microarray data (26). The most significantly enriched CM in component 1 was MODULE_47 (FDR = 6.55E−20) (Fig. 4*C*). This category included proteins such as ACTN1, BGN, COL1A1, COL1A2, COL6A1, COL6A2, COL6A3, COL6A4, FN1, LUM, POSTN, and SERPINH1 (Fig. 4*C*). There is a large overlap between these drivers and the reported prognostically favorable stromal-1 signature, reflecting extracellular matrix deposition (6). In fact, the stromal signature study showed that a multivariate model created from three gene expression signatures, germinal center B-cell (COO), stromal-1 (extracellular matrix deposition), and stromal-2 (tumor blood vessel density), was a better predictor of survival than the COO classification alone. Hence, survival of DLBCL patients after treatment is influenced by several biological attributes including the COO and the tumor microenvironment (6). In addition, expression levels of the ECM signature proteins we depicted in component 1 are on average higher in the GCB subtype. These findings confirm what has been reported previously (51) and show that our proteomic analysis captured the COO classification as well as other intrinsic biological differences between the subtypes.

##### Cancer-associated Characteristics of ABC-DLBCL Compared with GCB-DLBCL Subtypes

**Fig. 5.**
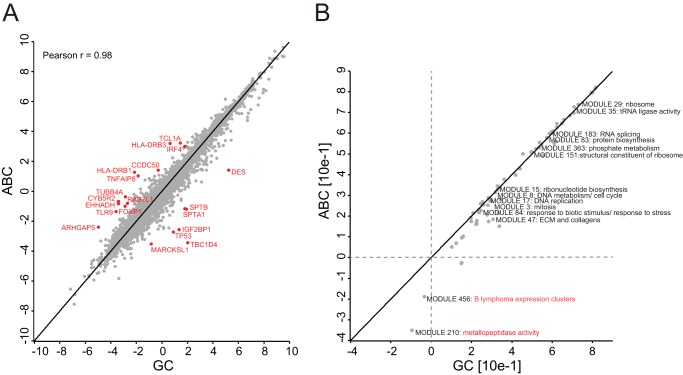
**ABC-DLBCL *versus* GCB-DLBCL.** *A*, Pearson correlation of ABC-DLBCL (*ABC*) *versus* GCB-DLBCL (*GC*) after taking median expression values of protein groups across patients in each subtype. *B*, two-dimensional annotation enrichment of ABC-DLBCL against GCB-DLBCL using cancer modules.

After assigning a subtype to each patient sample based on gene expression profiling classification, we treated the samples as biological replicates of the same disease entity. We grouped patients belonging to the same subtype together and calculated the median expression value for each protein group. The proteomes of GCB-DLBCL *versus* ABC-DLBCL had very high correlation (Pearson *r* = 0.98). Against this background of very high overall similarity, investigation of outliers from this tight cloud revealed markers that our unsupervised PCA had already indicated as well as novel candidate markers, which are connected to the known biology of the disease (Fig. 5*A*). This included TCL1A, FOXP1, and TLR9, which are up-regulated in the ABC subtype. In fact, both TCL1A and FOXP1 are immunohistochemical markers of adverse outcome in DLBCL (52, 53). FOXP1 was also reported to occur in a subgroup of non-GCB-DLBCLs (54), and TCL1A has been suggested as a tumor-associated antigen for immunotherapeutic strategies in common B-cell lymphomas (55). TLR9, another ABC-DLBCL-specific subtype candidate, is a toll-like receptor that senses microbial DNA containing unmethylated CpG sequences. It has recently been shown that lymphoma-associated mutations in MYD88 amplify the effects of upstream TLR9 activation rather than conferring autonomous NF-κB activation (56). This raises the possibility that nucleic acids in the tumor microenvironment drive the proliferation of these lymphomas (57).

Next, we performed a two-dimensional annotation enrichment analysis (27), which detects annotation terms whose members show consistent behavior in one or both of the data dimensions, in this case the ABC-DLBCL *versus* the GCB-DLBCL proteome. Here, we used CMs for deriving differential cancer-associated gene sets between these two closely related entities of DLBCL. As expected from the high proteome correlation, the subtypes are very similar in almost every cancer module annotated such as RNA splicing, protein biosynthesis, and DNA replication. However, MODULE_456, which corresponds to “B lymphoma expression clusters,” and MODULE_210, which corresponds to “metallopeptidase activity,” showed lower expression in the ABC subtype. MODULE_456 consists of 115 genes and is annotated to be significantly induced in B-cell lymphomas (*p* = 2.7E−05) and specifically in GCB-DLBCL (*p* = 3.0E−05). This confirms what we observed in our analysis (Fig. 5*B*). The metallopeptidase and metalloendopeptidase gene sets comprising MODULE_210 consist of 28 genes and were significantly induced in microarrays of DLBCL (*p* = 1.5E−06) and GCB-DLBCL (*p* = 5.1E−05) specifically (26). The proteins that we found in this gene set are particularly interesting given the role of matrix metalloproteinases (MMPs) in mediating tumor invasion.

The candidate differentially expressed proteins and categories clearly reflected relevant biological differences between ABC-DLBCL and GCB-DLBCL. However, these candidates cannot necessarily be used as markers of classification. For instance, the expression profiles of biologically interesting candidates like TCL1A and FOXP1 show a high degree of variability within each subtype (supplemental Fig. S2). More sophisticated statistical tools are required to achieve a panel of candidate proteins that can be used for diagnostic purposes as discussed in the next section.

##### Support Vector Machines Combined with Feature Selection

In clinical studies, tumor and host variability combined with the large feature space of the data set (thousands of proteins compared with a relatively small number of patients) makes it difficult to identify disease-relevant proteins. We addressed these challenges with a supervised learning method, SVMs, in combination with a test statistic-based feature selection strategy. SVMs are a well established machine learning technique that trains a predictor that best distinguishes between the known classes of the samples (in our case GCB and ABC lymphoma subtypes). The principle of an SVM predictor is the definition of a so-called separation hyperplane that segregates the subtypes as clearly as possible in a training data set, which can be a subset of the measured samples. Using this “machine-learned” hyperplane, new samples of unknown subtype can be classified as GCB or ABC depending on the side of the separation hyperplane on which each of these samples falls. The strength of SVMs lies in their ability to perform well in high dimensional data.

We combined the SVM-based prediction with feature selection to optimize the performance of the classifier and to identify strongly discriminative features or proteins. The feature selection method used *p* values from standard analysis of variance tests. As disease-relevant features that show large quantitative differences between the two subtypes are more easily detectable and thus are potentially clinically more relevant, we performed the ranking of the proteins such that it depended not only on the statistical significance of their differential expression between the different subtypes but also on the actual size of this difference. The advantage of this method is that proteins with low *p* values and high -fold change receive higher ranks than those with low *p* values and small -fold change.

**Fig. 6.**
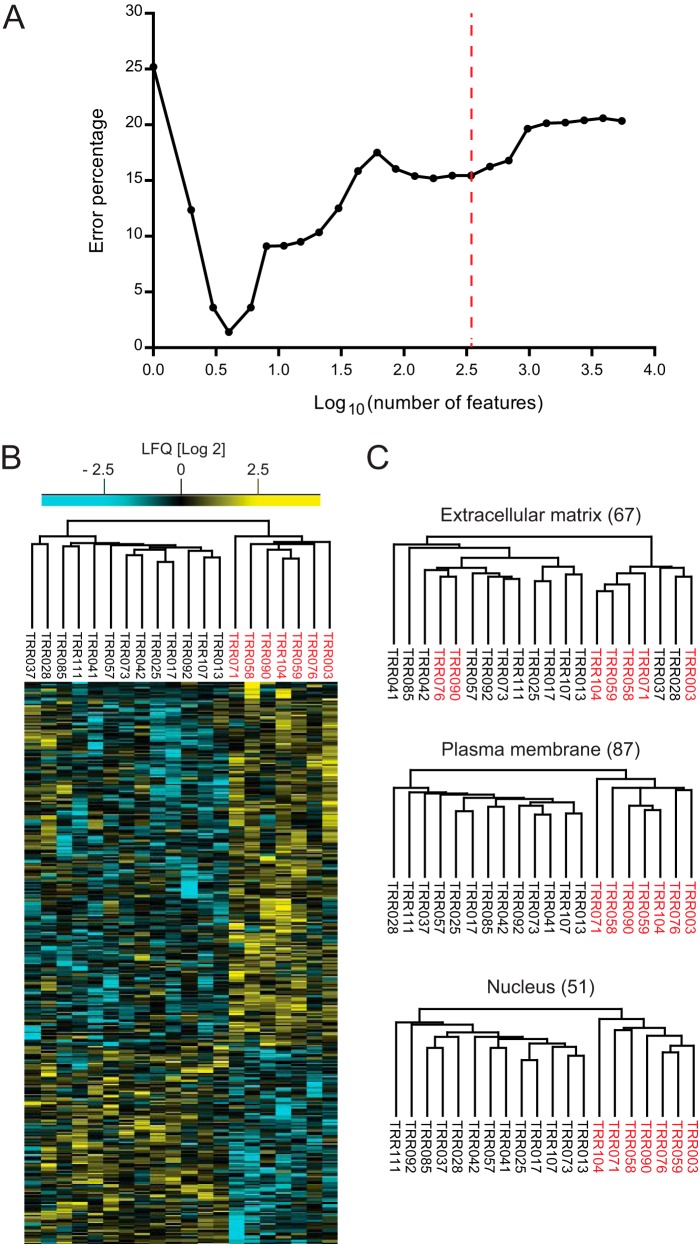
**Support vector machine analysis for optimal feature selection.** *A*, support vector machine feature selection using *p* values of standard analysis of variance tests resulted in a set of four features with 1.4% error. *B*, unsupervised hierarchical clustering of top 343 protein candidates or features determined by support vector machine analysis. *C*, unsupervised hierarchical clustering of extracellular matrix, plasma membrane, and nuclear proteins in the 343 top protein candidates.

Feature selection was embedded in a cross-validation procedure to avoid the problem of overfitting and wrong estimation of the performance of the classifier. In each iteration (total, 1,000) of a random sampling cross-validation, we used 90% of the data for training and feature ranking and the rest for testing and optimization of the number of features. The analysis resulted in a set of four ranked features that perform almost perfectly in the classification of the subtypes (1.4% error rate) (Fig. 6*A*). These top four candidates are TBC1D4, PALD1, TNFAIP8, and MME (CD10). The protein expression level of the four candidates is relatively stable across patient samples from the same subtype (supplemental Fig. S3). MME is part of previous immunohistochemistry-based classification algorithms (11, 58), and it was retrieved as a candidate in our *N*-glycoproteome cell line-based study (19). TBC1D4 plays a role in glucose uptake, TNFAIP8 is NF-κB regulated and involved in blocking apoptosis, and PALD1 is a newly studied protein that may play a role in tumor invasiveness and metastasis.

Next, we were interested in comparing ranked features with the digital gene expression (NanoString)-based test of 20 genes that has been recently published and put into use in a clinical trial (12). The model is composed of eight genes (*TNFRSF13B*, *LIMD1*, *IRF4*, *CREB3L2*, *PIM2*, *CYB5R2*, *RAB7L1*, and *CCDC50*) overexpressed in ABC-DLBCL, five housekeeping genes, and seven genes (*MME*, *SERPINA9*, *ASB13*, *MAML3*, *ITPKB*, *MYBL1*, and *SIPR2*) overexpressed in GCB-DLBCL. Different gene signatures of a disease often have little overlap in their constituent genes even when they are derived by the same technology. In light of this, it was reassuring that 30% of the differentiating genes in the RNA-based test (*IRF4*, *CYB5R2*, *RAB7L1*, *CCDC50*, and *MME*) were among the 17 top SVM-ranked features of our analysis (supplemental Table S2).

For a broader selection of differential features, we used as an error rate cutoff, the point beyond which the correct unsupervised hierarchical clustering of the subtypes was lost. This resulted in 343 features (Fig. 6*B*). Interestingly, upon filtering for ECM, nuclear, and plasma membrane proteins from these top 343 features, the last two categories maintained correct segregation on their own, reflecting the cell-of-origin classification (Fig. 6*C*).

Encouragingly, the top 343 features overlapped with 17 protein groups previously depicted by the 55-protein cell line signature with segregating power (supplemental Table S2). In addition, the set of 343 protein groups included 33 transcription factors, 14 protein kinases, and 12 oncogenes (supplemental Table S3, A and B). Upon dividing the 343 protein groups into their two main clusters (one relatively up-regulated in ABC-DLBCL and the other relatively up-regulated in GCB-DLBCL), we performed network analysis to investigate possible connections between them. Genes up-regulated in the ABC-DLBCL subtype highlighted the CARD11-PKCB signaling core (supplemental Fig. S4A) that drives NF-κB signaling upon B-cell receptor signaling (59). The GCB-DLBCL subtype showed an LCK-PAG-P2K signaling module (supplemental Fig. S4B), which has been shown to be oncogenic in other lymphomas (60). In addition to an ECM core that we previously depicted to be up-regulated on average in the GCB subtype, we also observe an MHC II network that has been previously reported to be on average higher in GCB (51).

##### Conclusions and Outlook

Previously, we had shown unambiguous segregation of patient-derived DLBCL cell lines into their COO subtypes based on their global protein expression profiles as well as an enriched set of membrane proteins (18, 19). In this study, we have analyzed 20 FFPE DLBCL patient samples, attaining a quantitative depth of more than 9,000 proteins, which to our knowledge is the largest lymphoma proteome available. Correct segregation of the subtypes based on their protein expression profiles was possible after applying a cell line-derived signature from our previous studies or by using the whole set of proteins quantified in at least 75% of the samples. When global protein expression profiles were used, the COO classification was not as clear-cut as in the cell lines. This is most likely due to increased complexity of this system in which several important biological signatures (extracellular matrix and MHC II) also influence segregation. In fact, these signatures are known to be very valuable in the overall prediction of survival in DLBCL patients (51). Our results clearly show that global expression proteomics can segregate cancer types based on tumor samples from patients. Importantly, for practical applications, our measurements only require small amounts of FFPE material, which is readily available in tissue banks or informal sample collections.

The high number of biologically relevant potential markers retrieved here underscores the potential of future applications of proteomics to clinical questions such as tumor segregation. Our analysis highlighted both the COO signature and the ECM signature in line with the “gold standard” predictor of survival, which includes the COO classification as well as stromal signatures (6, 34). Nuclear and membrane proteins reflect the COO, but the ECM signature is more likely reflecting mechanisms through which lymphoma cells interact with their environment. Hence, they are at least partly independent signatures, and patient survival depends on both.

In a classical view of biomarker development, global MS-based proteomics plays a role primarily in the discovery phase (61). In postdiscovery studies, MS-based or ELISA-based targeted approaches would then be used on specific signature proteins. However, it is interesting to speculate that an untargeted approach could also be used in this phase, which would have the advantage of not discarding valuable information contained in the patient samples. Considering the rate of MS developments, measuring a proteome of complex biological samples such as patient tissues comprehensive and accurate enough for tumor classification in a high throughput manner should be achievable in the near future. In addition, further improvements of sample preparation methods will allow easier sample handling and higher reproducibility (62). In conclusion, continuous MS-based technological advances hold great promise for future characterization and diagnosis of subtypes not only of B-cell lymphomas but any closely related tumor subtypes.

## Supplementary Material

Supplemental Data

## Footnotes

^1^ The abbreviations used are:

DLBCL: diffuse large B-cell lymphoma
ABC: activated B-cell-like
CLL: chronic lymphocytic leukemia
COO: cell-of-origin
FFPE: formalin-fixed paraffin-embedded
GCB: germinal center B-cell-like
PCA: principal component analysis
SILAC: stable isotope labeling with amino acids in cell culture
SVM: support vector machine
LFQ: label-free quantification
FASP: filter-aided sample preparation
CM: cancer module
FDR: false discovery rate
ECM: extracellular matrix
SDS: sodium dodecyl sulfate.
