# Supplementary material for: Machine Learning-based Classification of Diffuse Large B-cell Lymphoma Patients by Their Protein Expression Profiles
Source: Mol Cell Proteomics. 2015 Aug 26;14(11):2947–60. doi: 10.1074/mcp.M115.050245 (PMC4638038; doi:10.1074/mcp.M115.050245)
Supplement: Supplemental Data [file supp_14_11_2947__index.html]

Machine Learning Based Classification of Diffuse Large B-cell Lymphoma Patients by their Protein Expression Profiles — Machine Learning-based Classification of Diffuse Large B-cell Lymphoma Patients by Their Protein Expression Profiles — Subtyping of Lymphoma Patients Based on Their Proteomes — Supplemental Data 

# Machine Learning-based Classification of Diffuse Large B-cell Lymphoma Patients by Their Protein Expression Profiles

## Supplemental Data

- Supplementary figure 1 - Quantified proteomes of DLBCL patients employing the hybrid LFQ algorithm
- Supplementary figure 2 - Expression profiles of TCL1A and FOXP1 across patient samples
- Supplementary figure 3 - Expression profiles of PALD1, TBC1D4,TNFAIP8 and MME across patient samples
- Supplementary figure 4 - Network analysis of proteins upregulated in ABC-DLBCL and GCB-DLBCL
- Supplemental table S1 - Protein groups
- Supplemental table S2 - Overlap between top 343 SVM ranked proteins, cell line signature and RNA-based test
- Supplemental table S3 - Categories in top 343 SVM ranked proteins
