# Supplementary material for: Machine Learning-based Classification of Diffuse Large B-cell Lymphoma Patients by Their Protein Expression Profiles
Source: Mol Cell Proteomics. 2015 Aug 26;14(11):2947–60. doi: 10.1074/mcp.M115.050245 (PMC4638038; doi:10.1074/mcp.M115.050245)
Supplement: Supplemental Data [file supp_M115.050245_mcp.M115.050245-1.pdf]

Supplementary figure S1

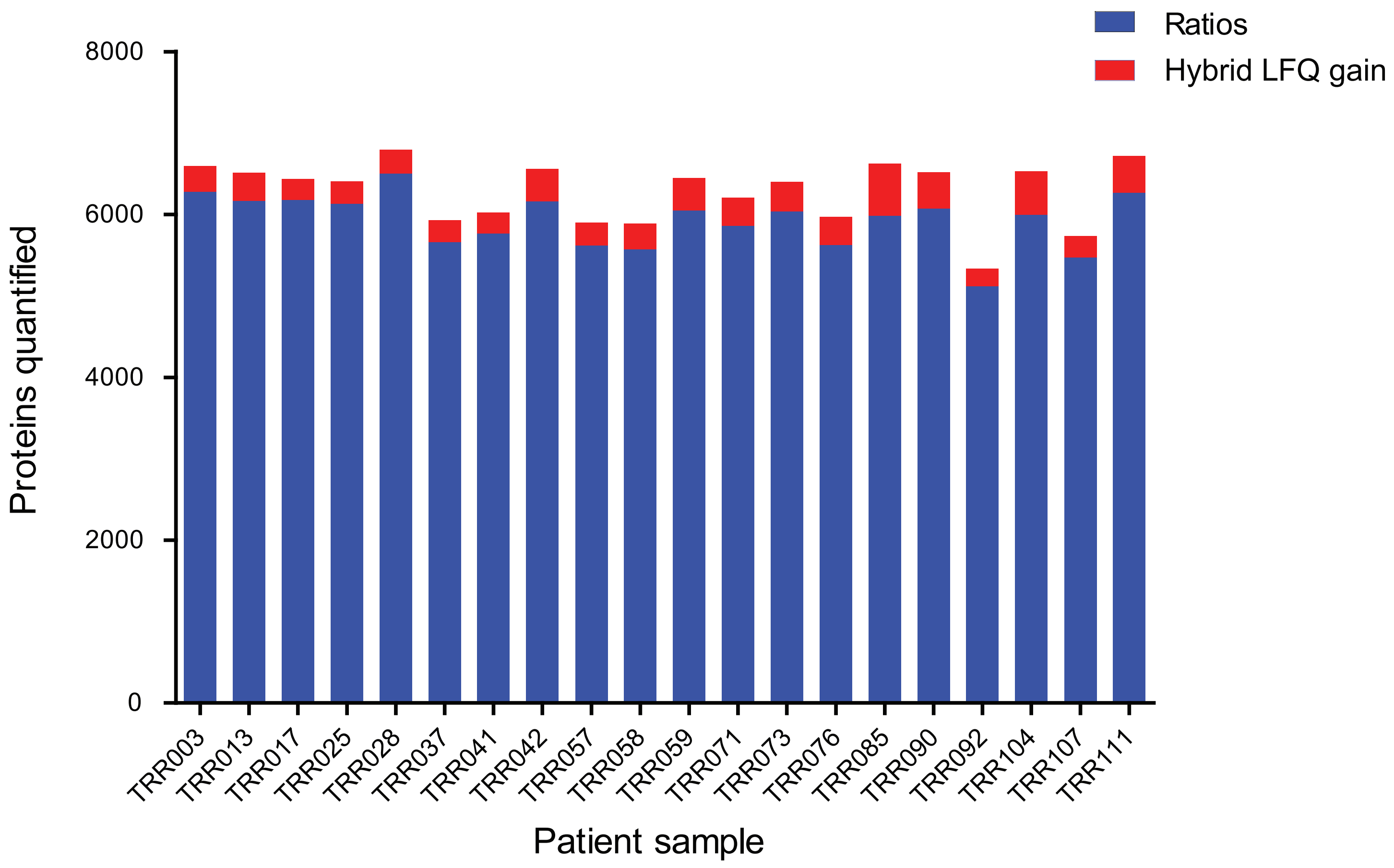

FIG. S1. Quantified proteomes of DLBCL patients employing the hybrid LFQ algorithm. Red bars indicate the gain from using the hybrid LFQ compared to using SILAC ratios alone.
