# Supplementary material for: Machine Learning-based Classification of Diffuse Large B-cell Lymphoma Patients by Their Protein Expression Profiles
Source: Mol Cell Proteomics. 2015 Aug 26;14(11):2947–60. doi: 10.1074/mcp.M115.050245 (PMC4638038; doi:10.1074/mcp.M115.050245)
Supplement: Supplemental Data [file supp_M115.050245_mcp.M115.050245-2.pdf]

Supplementary figure S2

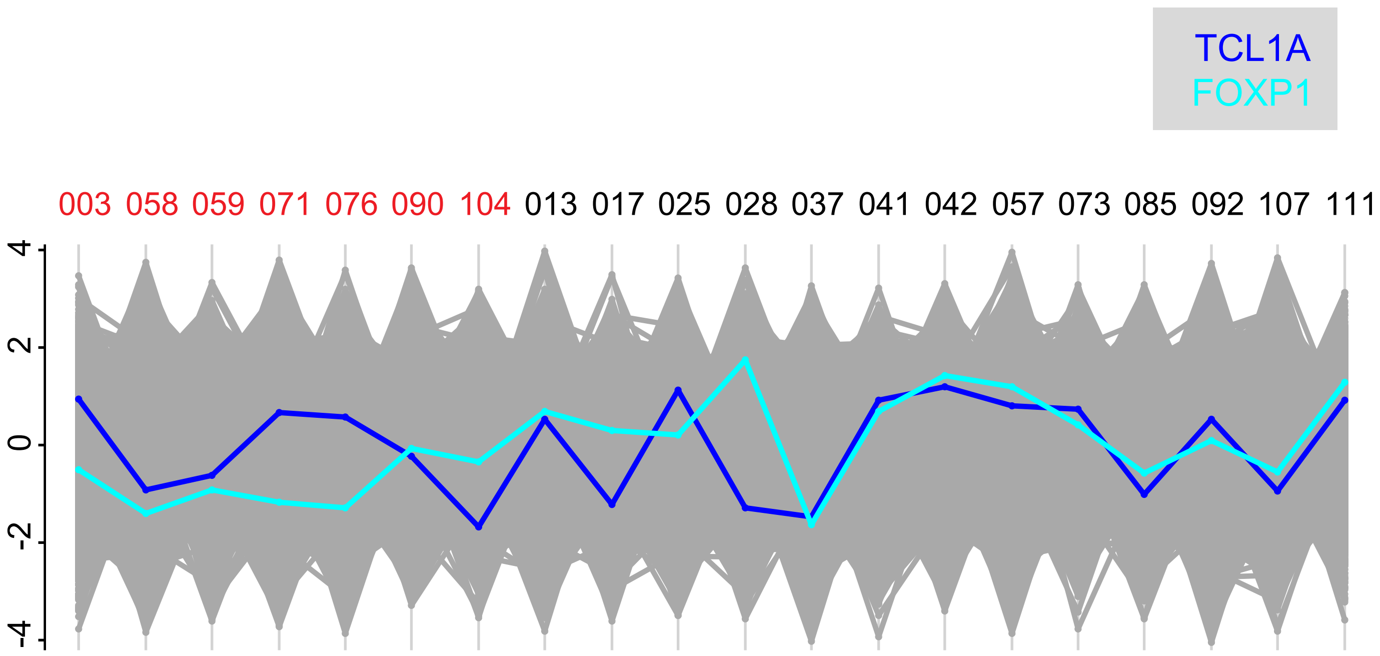

FIG. S2. Expression profiles of TCL1A and FOXP1 across patient samples. Patient samples highlighted in red correspond to GC-DLBCL patients and the ones highlighted in black correspond to ABC-DLBCL patients.
