# Supplementary material for: Machine Learning-based Classification of Diffuse Large B-cell Lymphoma Patients by Their Protein Expression Profiles
Source: Mol Cell Proteomics. 2015 Aug 26;14(11):2947–60. doi: 10.1074/mcp.M115.050245 (PMC4638038; doi:10.1074/mcp.M115.050245)
Supplement: Supplemental Data [file supp_M115.050245_mcp.M115.050245-3.pdf]

Supplementary figure S3

A

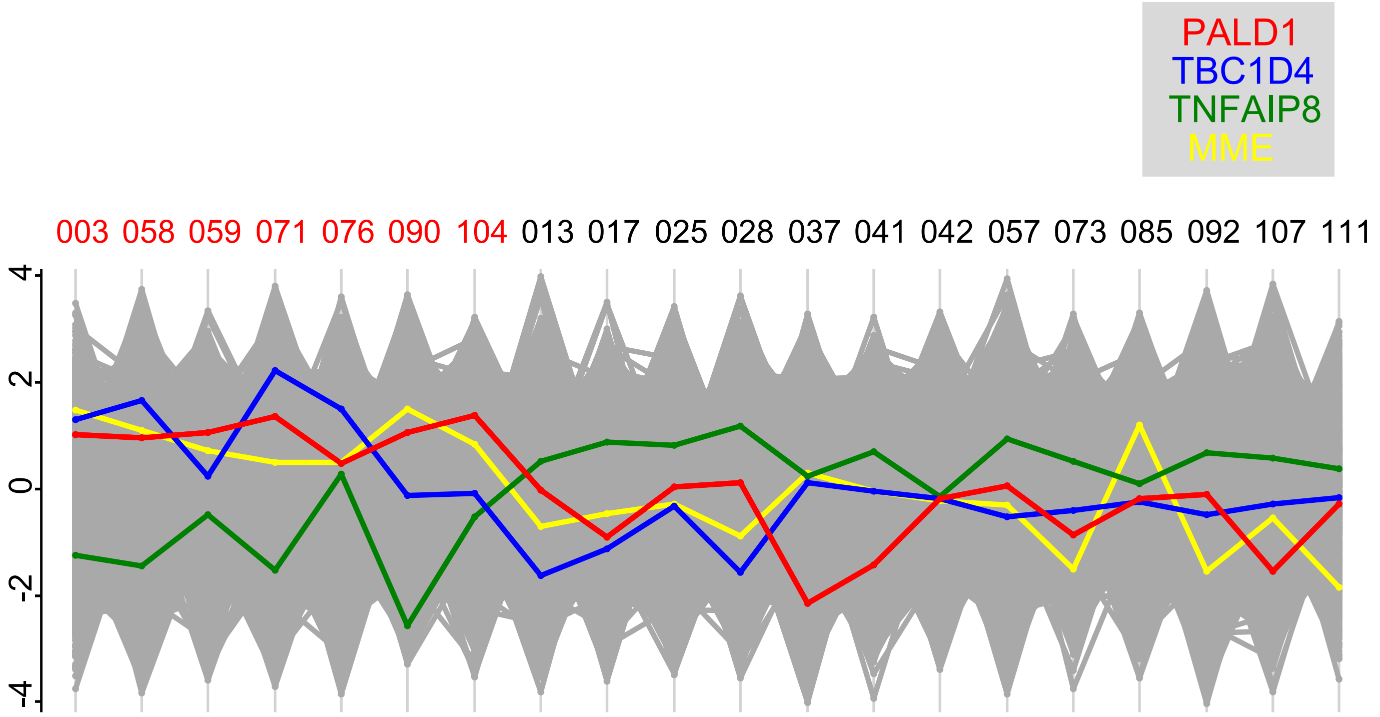

B

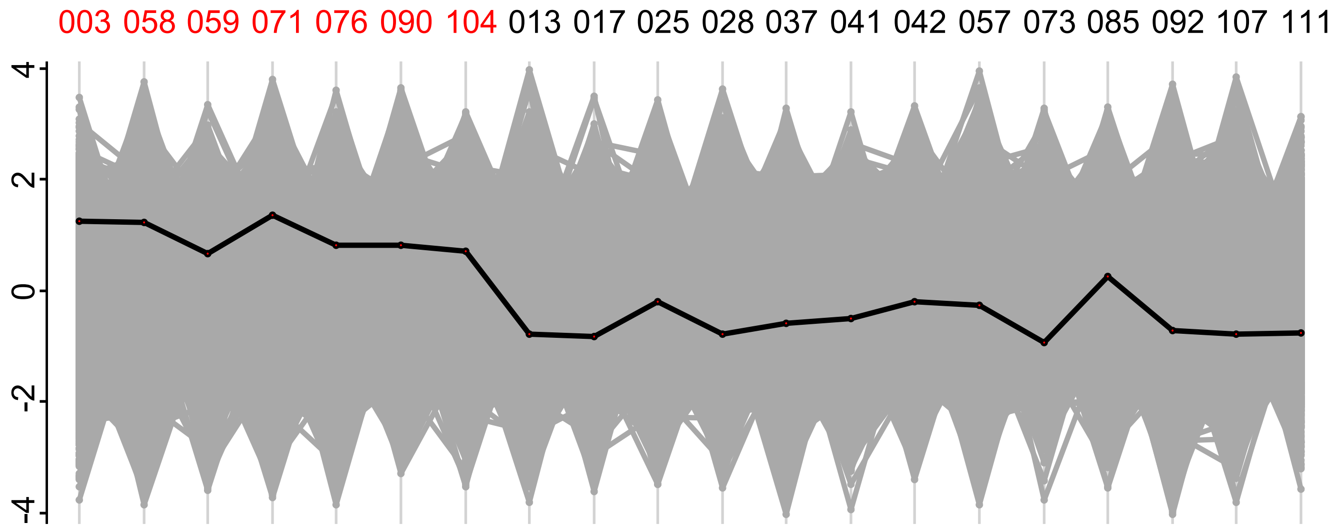

FIG. S3. A. Expression profiles of PALD1, TBC1D4,TNFAIP8 and MME across patient samples. Patient samples highlighted in red correspond to GC-DLBCL patients and the ones highlighted in black correspond to ABC-DLBCL patients. B. Average expression profile of PALD1, TBC1D4 and MME across patient samples.
