# Supplementary material for: Machine Learning-based Classification of Diffuse Large B-cell Lymphoma Patients by Their Protein Expression Profiles
Source: Mol Cell Proteomics. 2015 Aug 26;14(11):2947–60. doi: 10.1074/mcp.M115.050245 (PMC4638038; doi:10.1074/mcp.M115.050245)
Supplement: Supplemental Data [file supp_M115.050245_mcp.M115.050245-4.pdf]

Supplementary figure S4

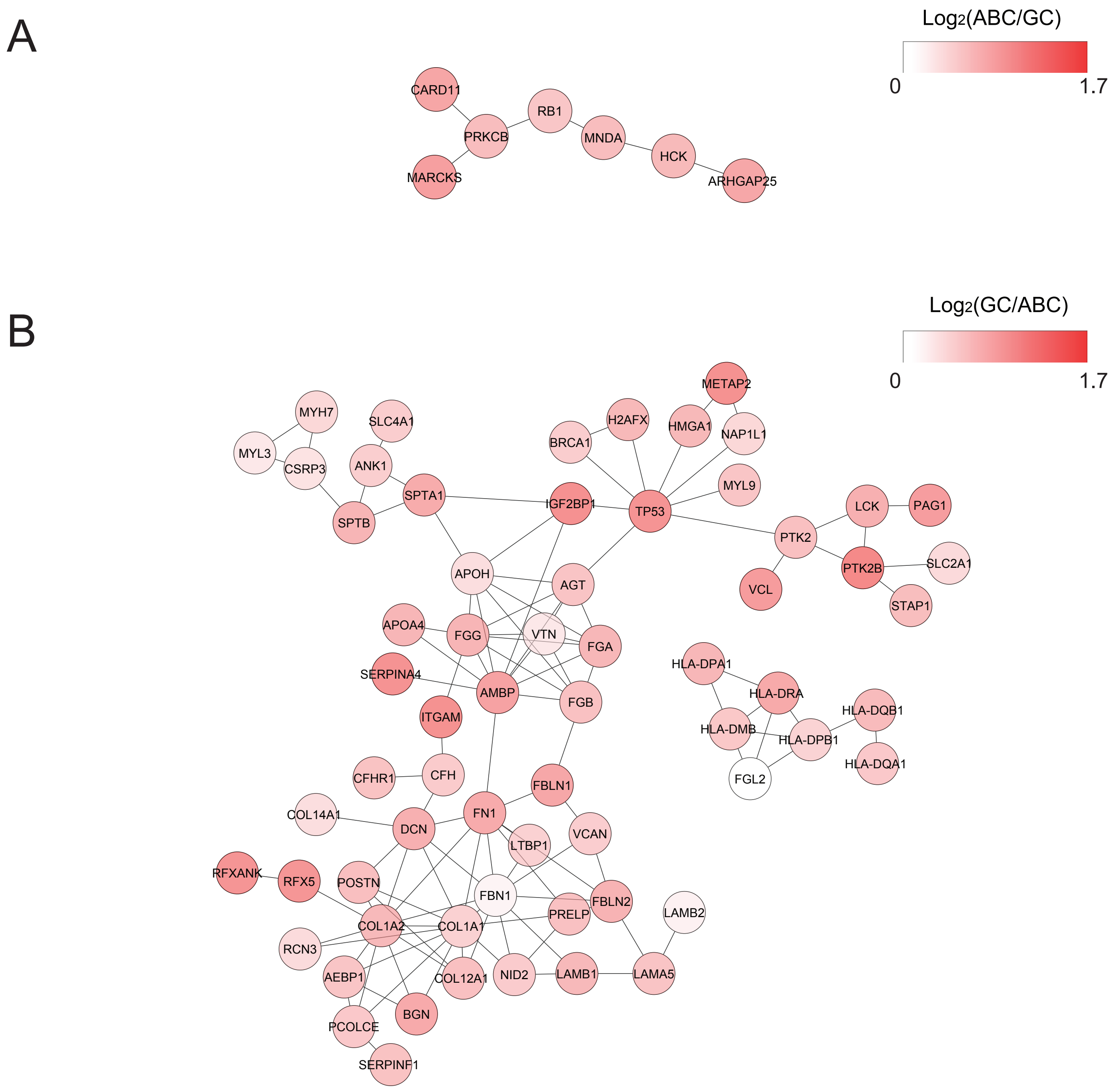

FIG. S4. Network analysis of proteins upregulated in ABC-DLBCL (A) and GCB-DLBCL (B).
