# Supplementary material for: Machine Learning-based Classification of Diffuse Large B-cell Lymphoma Patients by Their Protein Expression Profiles
Source: Mol Cell Proteomics. 2015 Aug 26;14(11):2947–60. doi: 10.1074/mcp.M115.050245 (PMC4638038; doi:10.1074/mcp.M115.050245)
Supplement: Supplemental Data [file supp_M115.050245_mcp.M115.050245-7.pdf]

Supplementary table S3

A

	Cytokines and growth factors	Transcription factors	Cell differentiation markers	Protein Kinases	Translocated cancer genes	Oncogenes	Tumor suppressors
Tumor suppressors	0	3	0	0	0	0	5
Oncogenes	0	4	0	2	10	12	
Translocated cancer genes	0	3	0	2	10		
Protein kinases	0	0	0	14			
Cell differentiation markers	0	0	11				
Transcription factors	0	33					
Cytokines and growth factors	3						

B

**Cytokines and growth factors**	**Transcription factors**		**Cell differentiation markers**	**Protein Kinases**	**Translocated cancer genes**	**Oncogenes**	**Tumor suppressors**
AGT	BATF	KAT5	ANPEP	CAMK4	BCL10	BCL10	BRCA1
IL16	BTAF1	LIMA1	BST2	CDK6	BCL2	BCL2	ERCC3
LTBP1	CHD1	LMO7	CD22	HCK	CDK6	CARD11	PMS1
	CHD2	MED17	CD44	IKBKE	COL1A1	CDK6	RB1
	CNPY3	MED23	CD9	LCK	EML4	COL1A1	TP53
	CRIP2	MNDA	ENTPD1	LYN	ERC1	EML4	
	CSRP3	NCOR2	F11R	MAP4K2	FOXP1	ERC1	
	DPF2	NFKBIB	ITGAM	PDK1	HMGA1	FOXP1	
	ERCC3	RB1	MME	PDK3	IRF4	HMGA1	
	ETS1	REL	SLC4A1	PRKCB	LCK	IRF4	
	FHL1	RELB	TLR9	PTK2		LCK	
	FOXP1	RFX5		PTK2B		REL	
	HLTF	RFXANK		SLK			
	HMGA1	SMAD5		TTN			
	IGHMBP2	SUB1					
	IRF4	TP53					
		ZNF318					
